# Dissemination of *Strongyloides stercoralis *in a patient with systemic lupus erythematosus after initiation of albendazole: a case report

**DOI:** 10.1186/1752-1947-2-156

**Published:** 2008-05-14

**Authors:** Catherine J Hunter, Mikael Petrosyan, Morris Asch

**Affiliations:** 1 Harbor UCLA Medical Center, W Carson Street, Department of Surgery, Torrance, CA 90502, USA; 2 University of Southern California, Keck School of Medicine, North State Street, Los Angeles, CA 90033, USA

## Abstract

**Introduction:**

*Strongyloides stercoralis *infection affects hundreds of millions of people worldwide. As immigration rates and international travel increase, so does the number of cases of strongyloidiasis in the United States. Although described both in immigrant and in immunosuppressed populations, hyperinfection and dissemination of *S. stercoralis *following the initiation of antiparasitic medication is a previously unreported phenomenon.

**Case presentation:**

Here we describe the case of a 38-year-old immunocompromised woman with systemic lupus erythematosus, who developed disseminated disease following treatment with albendazole (400 mg every 12 hours). Notably the patient was receiving oral prednisone (10 mg once daily), azathioprine (50 mg twice daily), and hydroxychloroquine (400 mg daily) at the time of hospitalization. The patient was subsequently treated successfully with ivermectin (200 mcg/kg daily).

**Conclusion:**

The reader should be aware that dissemination of *S. stercoralis *can occur even after the initiation of antiparasitic medication.

## Introduction

*Strongyloides stercoralis *is a nematode that infects approximately 100 million humans worldwide each year. Infection is endemic in tropical regions and may occur throughout South America, the Caribbean, Africa, and Europe [1] as well as the southern United States [2]. As international travel and immigration rates rise, so does the number of cases of strongyloidiasis within the United States. In fact, *S. stercoralis *can persist for many years without any apparent symptoms in individuals who have visited an endemic area [3]. Currently, the prevalence of *S. stercoralis *carriage in certain Northern American states has been reported to be as high as 3% of the population [2].

The life cycle of *S. stercoralis *in humans begins when free-living infective filariform larvae penetrate the skin and migrate hematogenously to the lungs [4]. Once the larvae reach lung capillary beds, they migrate through the capillary walls into the alveolar air spaces. The larvae are coughed up to the larynx, where they are swallowed, and thus gain access to the duodenum and jejunum. The larvae develop into adult females, which lay eggs that hatch non-migratory (rhabditiform) larvae that penetrate the mucosa, leading to internal auto-infection.

This auto-infective cycle may persist and dissemination has been reported due to immunocompromised status from HIV, chemotherapy, or corticosteroid therapy [5-7]. Corticosteroids are widely used in the management of systemic lupus erythematosus (SLE), and disseminated strongyloidiasis is reported after corticosteroid administration for this disease [8]. Dissemination may involve gut, stomach, lung and/or cerebrospinal fluid [9,10]. Furthermore, larval penetration of the intestinal wall during dissemination may result in bacteremia due to the introduction of bowel flora.

It is generally accepted that, without prompt treatment, hyperinfection may prove fatal. Here we describe the case of a patient who developed disseminated disease after corticosteroid treatment for SLE despite treatment with albendazole. The patient only showed improvement after institution of ivermectin.

## Case presentation

A 38-year-old woman emigrated from the Dominican Republic 1 year prior to presentation with complaints of 6 days of abdominal pain and blood-flecked emesis. Of note she had recently been diagnosed with SLE, and was undergoing treatment with oral prednisone (10 mg once daily), azathioprine (50 mg twice daily), and hydroxychloroquine (400 mg daily).

Physical examination revealed a thin woman with cushingoid features in no acute distress. Vital signs demonstrated a normothermic, normotensive patient with mild tachycardia. Abdominal examination was notable for epigastric tenderness and guaiac positive stool. Her skin was noted to have a diffuse erythematous reticular rash extending from her abdomen to her upper legs. Laboratory findings demonstrated mild thrombocytopenia (120,000 platelets/mm^3^), a white blood cell count of 13,000/mm^3^, with an automatic differential of 79.5% neutrophils and 1.1% eosinophils. Chest X-ray was within normal limits without pulmonary infiltrates. Her urine culture subsequently grew *Klebsiella pneumoniae*, and she was treated with ciprofloxacin. Both azathioprine and celecoxib were discontinued at time of admission.

**Figure 1:**
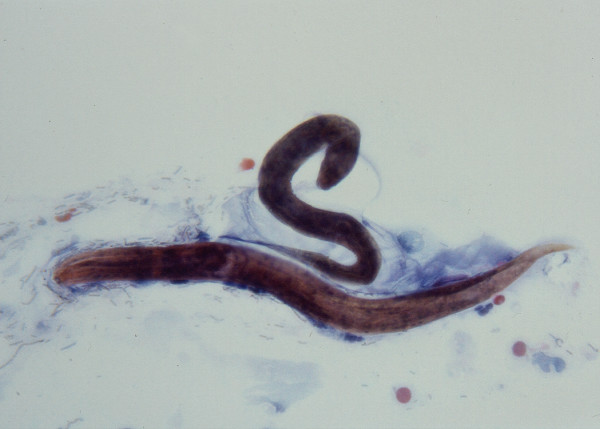
Esophageal brushing revealing the larval form of *Strongyloides stercoralis*.

**Figure 2:**
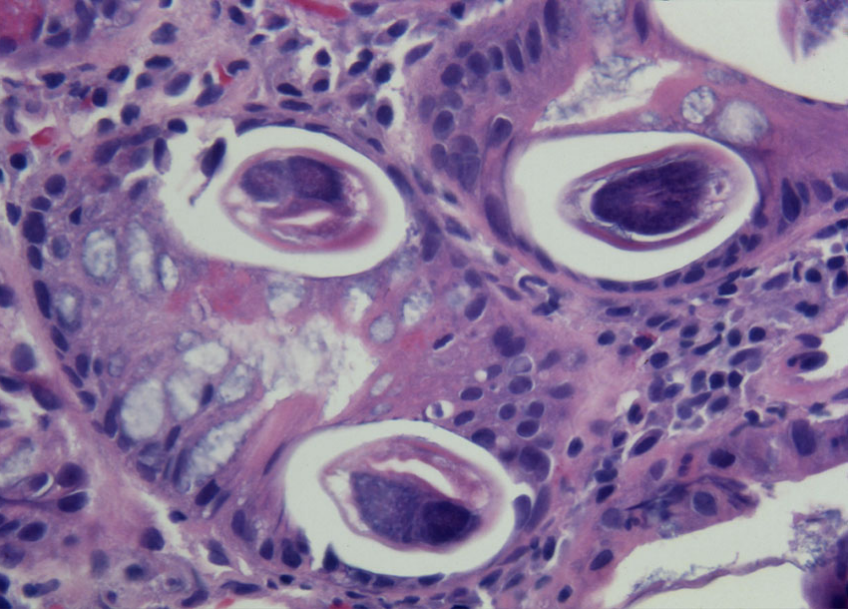
**Duodenal biopsy**. Multiple larval forms of *Strongyloides stercoralis in situ*.

The patient underwent upper endoscopy that revealed mild esophagitis and duodenitis. Esophageal brushings (Figure 1) and a duodenal biopsy (Figure 2) were collected which demonstrated *S. stercoralis*. Serial stool samples were collected and were subsequently noted to contain *S. stercoralis*. Serology testing by enzyme-linked immunoassay further confirmed the diagnosis.

Treatment with oral albendazole (400 mg twice daily) was initiated within 20 hours of presentation; however, the patient continued to experience abdominal discomfort. The truncal reticular rash also persisted despite therapy. Four days after admission, and 3 days after initiation of albendazole therapy, the patient developed respiratory distress, high fever, and hypotension. New pulmonary rales were audible over both lung fields and a chest radiograph demonstrated new diffuse opacities. Blood cultures and urine cultures were obtained. The patient was transferred to the intensive care unit where she was resuscitated with intravenous fluids, and received stress dose steroids. Her antibiotic coverage was broadened to include ciprofloxacin, metronidazole, vancomycin, and gentamicin, and her antiparasitic medication was changed to ivermectin (200 mcg/kg once daily). Blood cultures were positive for *Klebsiella pneumoniae*, *Enterococcus faecalis*, and *Escherichia coli*.

After 10 days of ivermectin and consistently negative stool examination for ova and parasites, antiparasitic therapy was discontinued. The patient was continued on appropriate antibiotics for 14 days and discharged home after a total of 22 days of hospitalization. The patient's serology tests had returned to normal by her 4-month follow-up visit.

## Discussion

Typically, hyperinfection syndrome occurs in patients from endemic areas of *S. stercoralis *who receive immunosuppressive therapy and present with polymicrobial sepsis. The diagnosis in such patients may at times be difficult because of a lower incidence of eosinophilia. Diagnosis by a single stool sample may fail to yield a diagnosis, since the detection rate is cited as 25% [11]. In our patient, 100% of stool samples were positive prior to therapy and during treatment with albendazole, possibly because of a high parasitic burden. Infection may also be diagnosed by serology, and can be followed-up to confirm successful treatment. Typically, serology will be negative within 6 months of *S. stercoralis *eradication. Our patient had normal serology 4 months after completion of therapy.

This case is unusual because disseminated disease occurred 3 days after initiation of therapy with albendazole. We are uncertain why dissemination occurred in this time sequence. A possible explanation includes albendazole-resistant *S. stercoralis*. Data suggest that regional differences already exist in albendazole susceptibility in a variety of nematodes [12]. Albendazole has a tendency to produce less tolerable side-effect profiles than ivermectin. Poor tolerance of albendazole by our patient may have led to malabsorption of albendazole (but not ivermectin). Randomized trials comparing ivermectin with albendazole and other antihelminths found ivermectin to be successful in eradicating larval forms [13]. Other possible explanations include a delayed response to therapy or induction of an inflammatory response that resulted in tissue damage and dissemination. Ivermectin may be superior to albendazole because of a cidal action on both the larval and adult forms of *S. stercoralis *[14,15].

The higher rate of hyperinfection in immunosuppressed patients receiving corticosteroids is not well understood. In addition to the broad immunosuppressive effect of corticosteroids, it has been observed in an animal model of strongyloides that female worms produce more eggs in the presence of exogenous steroids. This may further facilitate worm growth and development [16].

## Conclusion

Clinicians should be aware that the *S. stercoralis *hyperinfection syndrome may occur several days into appropriate antihelminth therapy and should remain vigilant for signs of sepsis even during the early days of therapy. Our findings are based on a single case report, and to better compare the utility of albendazole and ivermectin in the treatment of *S. stercoralis *hyperinfection syndrome, a randomized prospective trial would be required.

## Competing interests

The authors declare that they have no competing interests.

## Authors' contributions

CJH obtained the images and wrote the manuscript. MA and MP contributed significantly to the writing of this manuscript. All authors read and approved the final manuscript.

## Consent

Written informed consent was obtained from the patient for publication of this case report and accompanying images. A copy of the written consent is available for review by the Editor-in-Chief of this journal.
